# Simultaneous Image Reconstruction and Element Decomposition for Iodine Contrast Agent Visualization in Multienergy Element-Resolved Cone Beam CT

**DOI:** 10.3389/fonc.2022.827136

**Published:** 2022-02-01

**Authors:** Chao Wang, Hyunuk Jung, Ming Yang, Chenyang Shen, Xun Jia

**Affiliations:** ^1^ Innovative Technology of Radiotherapy Computation and Hardware (iTORCH) Laboratory, Department of Radiation Oncology, University of Texas Southwestern Medical Center, Dallas, TX, United States; ^2^ Department of Radiation Oncology, University of Texas Southwestern Medical Center, Dallas, TX, United States

**Keywords:** cone beam CT, radiotherapy, image guidance, liver cancer, multienergy cone beam CT, element decomposition

## Abstract

Iodine contrast agent is widely used in liver cancer radiotherapy at CT simulation stage to enhance detectability of tumor. However, its application in cone beam CT (CBCT) for image guidance before treatment delivery is still limited because of poor image quality and excessive dose of contrast agent during multiple treatment fractions. We previously developed a multienergy element-resolved (MEER) CBCT framework that included x-ray projection data acquisition on a conventional CBCT platform in a kVp-switching model and a dictionary-based image reconstruction algorithm that simultaneously reconstructed x-ray attenuation images at each kilovoltage peak (kVp), an electron density image, and elemental composition images. In this study, we investigated feasibility using MEER-CBCT for low-concentration iodine contrast agent visualization. We performed simulation and experimental studies using a phantom with inserts containing water and different concentrations of iodine solution and the MEER-CBCT scan with 600 projections in a full gantry rotation, in which the kVp level sequentially changed among 80, 100, and 120 kVps. We included iodine material in the dictionary of the reconstruction algorithm. We analyzed iodine detectability as quantified by contrast-to-noise ratio (CNR) and compared results with those of CBCT images reconstructed by the standard filter back projection (FBP) method with 600 projections. MEER-CBCT achieved similar contrast enhancement as FBP method but significantly higher CNR. At 2.5% iodine solution concentration, FBP method achieved 170 HU enhancement and CNR of 2.0, considered the standard CNR for successful tumor visualization. MEER-CBCT achieved the same CNR but at ~6.3 times lower iodine concentration of 0.4%.

## 1 Introduction

Image guidance is a critical component of cancer radiation therapy. By imaging the patient anatomy at the treatment position, image guidance allows accurate positioning of the tumorous target against the therapeutic radiation beam, thereby increasing dose to the target and sparing dose to nearby healthy organs. Clinical advantages of image guidance in radiotherapy have been previously demonstrated by a number of studies (1).

Cone beam CT (CBCT) installed on a medical linear accelerator (LINAC) is currently the most widely used tool for imaging guidance in radiation therapy (2). Yet, one challenging context of tumor visualization is liver cancer. Due to low soft-tissue contrast in x-ray CT and similar x-ray attenuation properties between liver tumor and normal liver, it is difficult to directly visualize liver tumor in CBCT, leading to tumor targeting uncertainty. This problem is further exacerbated by other issues such as imaging artifacts due to respiration-induced organ motion during image acquisition. Intravenous contrast enhancement (IVC) is routinely used in treatment planning CT of liver cancer radiotherapy because of improved tumor contrast. However, it cannot be reliably employed on standard CBCT due to poor image quality. A previous study found that IVC-CBCT was effective in only 1/4 of patients with tumor size larger than 120 cm^3^ under breath-hold scans. Small tumors or free-breathing CBCT do not show contrast enhancement (3). The dose of repeated injection of the iodine contrast agent over multiple treatment fractions further leads to the concern of toxicity. Hence, there is a strong desire to improve CBCT technology to support image guidance in liver cancer radiotherapy. This is particularly needed in adaptive radiotherapy to enable accurate tumor delineation at each fraction (4, 5).

One potential approach to increase sensitivity of CBCT imaging to imaging contrast agent for tumor visualization is to take advantage of energy dimension of the x-ray imaging. As the energy dependence of x-ray attenuation property of iodine is different from other materials in a human body, such as tissue and bone, it may be possible to differentiate iodine contrast from other materials by utilizing information provided by the energy dimension. In fact, extensive studies have been conducted on the CT platform to realize dual-energy or multienergy CT function and to differentiate materials, with iodine imaging being one of the major applications (6–9). On the CBCT platform side, Zbijewski et al. (10) studied the accuracy of material classification in dual-energy CBCT under different reconstruction algorithms using a table-top system. Lee et al. (11) realized the single-scan dual-energy CBCT function using a multislit filter installed between the x-ray source and the scanned object. The filtered and unfiltered x-ray beams generated projection data at two different energy levels. A novel reconstruction algorithm was developed to employ the joint sparsity between the low- and high-energy CT images. In another study, Li et al. (12) developed a reconstruction algorithm that utilized spatial and spectral correlation among images to improve image quality. Lately, Cassetta et al. (13) achieved fast-kilovoltage peak (kVp) switching function for dual-energy CBCT using the on-board imager of a commercial LINAC and successfully produced virtual monoenergetic and relative electron density images.

In our previous study, Shen et al. (14) successfully controlled the CBCT platform on a commonly used LINAC to realize multienergy CBCT data acquisition *via* the kVp switching scheme, i.e., taking x-ray projections with the kVp level cycling through different levels among projections. To address the undersampling problem caused by the kVp switching, they also developed a multi-energy element-resolved (MEER) CBCT framework to simultaneously reconstruct CBCT images at different kVp levels, as well as electron density map and the maps of a few major chemical elements. A physics model that correlates the CBCT images at different kVp levels, the electron density image, and the element images was built into the reconstruction algorithm, which served as a strong constraint on the solution to ensure their quality. In this paper, we report our recent developments to investigate the feasibility of using the MEER-CBCT framework for iodine contrast agent identification. The contributions of this study are twofold. First, on the algorithm side, we extended the previous MEER-CBCT reconstruction algorithm Shen et al. (14) to include iodine-related materials in the dictionary of the algorithm, which allowed us to handle the image reconstruction problem in the presence of the iodine contrast agent. We further converted the nonconvex optimization model in our previous study (14) into a convex form, making it easier and more efficient to solve the problem. Second, we also performed comprehensive simulation and experimental studies to quantitatively determine the lowest possible contrast agent concentration level identifiable in MEER-CBCT images to demonstrate the potential feasibility of this method for iodine contrast imaging.

## 2 Methods

### 2.1 Image Reconstruction Model

The image reconstruction model generally followed that in our previous study (14). In this study, we converted the model into a convex form to make it numerically more tractable. We also included materials with iodine in the dictionary to handle the reconstruction problem in the presence of the contrast agent.

The general form of MEER-CBCT model can be formulated as follows:


(1) $$\underset{F,\rho \geq 0,\lambda \geq 0}{\min}\frac{1}{2}R(F)+\frac{\beta }{2}G(F,\rho ,\lambda ).$$

The first term *R(F)* is established for ME-CBCT reconstruction problem, where *F* = [*f*_1_, *f*_2_, … , *f_N_*]∈ *R^M^* ^×^ *^N^* denotes black CBCT images at *N*=3 different energy channels. *f_i_* ∈ *R^M^* represents an image of the x-ray attenuation coefficient at the *i*th energy channel. *M* is the number of pixels in the image.

The second term *G*(*F*, *ρ*, *λ*) characterizes the relationship among *F*, the image of relative electron density (rED) to water *ρ* ∈ *R^M^* ^×^ *^M^*, a diagonal matrix with each diagonal element corresponding to rED of each voxel, and images of elemental compositions (EC) *λ* ∈ *R^M^* ^×^ *^D^*, where *D* indicates the number of elements of interest. In this study, we focused on three elements (*D*=3), i.e., hydrogen (H), oxygen (O), and iodine (I). The first two are major elements constructing majority of human nonbone tissues, and I is the effective element of the iodine contrast agent. We did not consider bony tissues, because this study focused on iodine contrast identification and it is expected that iodine contrast agent mainly exists in soft tissues. *β* is the parameter balancing the contributions from the two terms. Note that *ρ* ≥ 0 and λ ≥ 0 were naturally requested. In addition, let 1*_D_* and 1*_M_* denote column vectors of length *D* and *M* with all entries being in unity, λ1*_D_* = 1*_M_* was required, since the compositions of all elements in each voxel should add up to the unity.

#### 2.1.1 Image Reconstruction

We considered a tight-frame-based image reconstruction model (15, 16) in this study. As such, the detailed expression of *R(F)* can be given as


(2) $$R(F)={\left||PF-B|\right|}_{F}^{2}+2\alpha_{1}\sum_{i=1}^{N}{\left||Wf_{i}|\right|}_{1},$$

where *P* indicates the x-ray projection matrix characterizing the CBCT data acquisition process, while *B* represents the acquired CBCT projection data. Note that in a kVp switching data acquisition, images at different kVp levels were projected to different angles. This projection angle information was implicitly contained in the projection matrix *P*. In this model, α_1_ is the regularization parameter, and *W* is the tight frame (TF) operator. ||⋅||*_F_* and ||⋅||_1_ denote the matrix Frobenius norm and *l*_1_ norm, respectively. The first term in Eq. (2) enforced fidelity between the reconstructed CBCT images and acquired projections. The second term encouraged sparsity in the TF-transformed images to help in removing noise and artifacts while preserving edges in *F*.

#### 2.1.2 Material Decomposition

In Eq. (1), *G*(*F*, *ρ*, *λ*) was established to relate the x-ray attenuation coefficients with rED and EC. This was achieved based on an empirical model (17):


(3) $$f_{i}=\rho (k_{i}^{PE_{\tilde{Z}}3.62}+k_{i}^{R_{\hat{Z}}1.86}+k_{i}^{C}).$$

Here, $k_{i}^{PE},k_{i}^{R}\text{ and }k_{i}^{C}$ are parameters characterizing contributions of photoelectric effect, Rayleigh scattering, and Compton scattering, respectively, to the x-ray attenuation at the *i*th energy level. These parameters are dependent on the specific CBCT scanner. For a given scanner, they can be obtained *via* a calibration process (18). In addition, *λ* was encoded in $\tilde{z}\text{ and }\hat{z}$ as $\tilde{z}={\left(\lambda z^{3.62}\right)}^{\frac{1}{3.62}}\text{ and }\hat{z}={\left(\lambda z^{1.86}\right)}^{\frac{1}{1.86}},$ where *z* = [*z*_1_, *z*_2_, …, *z_D_*]*^T^* ∈ *R^D^* ^×1^ gives the atomic numbers for *D* different elements. Furthermore, let $K_{M}^{C}=1_{M}(k_{1}^{C},k_{2}^{C},\ldots ,k_{N}^{C})$ , and *K* = (*k*_1_, *k*_2_, …, *k_N_*) where $k_{i}=k_{i}^{PE_{Z}3.62}+k_{i}^{R_{Z}1.86}$ Through a simple derivation, the forward model in Eq. (3) can be rewritten into a matrix equation form for multiple energy levels, i.e.,


(4) $$F=\rho (\lambda K+K_{M}^{C}).$$

We further assumed that EC of each voxel can be sparsely represented over a dictionary consisting of EC of different tissues. Hence, we expressed EC as λ ≈ *V*Λ, where *V* ∈ *R^M^* ^×^ *^E^* gives the dictionary coefficients with each row being a sparse vector specifying the contribution of each dictionary material. This leads to a model $F=\rho (V\Lambda K+K_{M}^{C})$ .

With this equality connecting EC, rED with x-ray attenuation image, it would be straightforward to define an objective function as the difference between the two sides of this equation, $||F-\rho (V\Lambda K+K_{M}^{C})|{|}_{F}^{2}$ , and minimize it in the reconstruction problem. The requirement that the representation over Λ is sparse can be achieved by minimizing an objective function term of ||*V*||_0_, where ||⋅||_0_ denotes the *l*_0_ norm.

However, one obvious limitation of this approach is the nonconvex form of the objective function due to the product of *ρ* and *v*, as well as the *l*_0_ norm. To circumvent the problem, we combined *ρ* and *v* and defined a new variable *X* = *ρ⋅V*. Since it was required that λ1*_D_* = 1*_M_*, it followed that *V*1*_E_* = *V*Λ1*_D_* = 1*_M_*, where 1*_E_* is the column vector of length *E* with all elements being 1. We also relaxed the sparse penalty from the *l*_0_ form to the convex *l*_1_ form. With these changes, the objective function for the material decomposition can be expressed as


(5) $$G(F,\rho ,v)=||F-X(\Lambda K+K_{E}^{C})|{|}_{F}^{2}+\frac{2\alpha_{2}}{\beta }\sum_{j=1}^{M}||x_{j}|{|}_{1},$$

where $K_{E}^{C}=1_{E}(k_{1}^{C},k_{2}^{C},\ldots ,k_{N}^{C})$ . *x_j_* ∈ℝ*^M^* ^× 1^ is the *j*th row of *x* and α_2_ is the sparsity regularization parameter.

#### 2.1.3 Combined Model

With the image reconstruction and material decomposition terms defined in Eq. (2) and Eq. (5), the complete convex model of MEER-CBCT reconstructoin can be written as:


(6) $$\begin{array}{c} \{F^{*},X^{*}\}=\arg\min_{F,X\geq 0}\frac{1}{2}||PF-B|{|}_{F}^{2}+\frac{\beta }{2}||F-X(\Lambda K+K_{E}^{C})|{|}_{F}^{2}\\ +\alpha_{1}\sum_{i=1}^{N}||Wf_{i}|{|}_{1}+\alpha_{2}\sum_{j=1}^{M}||x_{j}|{|}_{1}. \end{array}$$

After we solve this problem to obtain *x*, we can easily compute *ρ* and *v* as


(7) $$\rho =\text{diag}(X1_{E}),\text{ and }V=\rho^{-1}X,$$

### 2.2 Numerical Algorithm

The model in Eq. (6) is convex and can be solved by the Alternating Direction Method of Multipliers (ADMM) (19). Similar to the iterative scheme proposed in (14), we incorporated ADMM to split the optimization problem into several subproblems and solved *F* and *x* in subproblems in an alternating fashion. Specifically, after introducing auxiliary variable, the augmented Lagrangian function was


(8) $$\begin{array}{c} L(F,X,U,\eta_{1})=\frac{1}{2}{\left||PF-B|\right|}_{F}^{2}+\frac{\beta }{2}{\left||F-X(\Lambda K+K_{E}^{C})|\right|}_{F}^{2}\\ +\alpha_{1}\sum_{i=1}^{N}{\left||u_{i}|\right|}_{1}+\alpha_{2}\sum_{j=1}^{M}{\left||x_{i}|\right|}_{1}+I_{+}(X)\\ +\frac{\mu_{1}}{2}{\left||U-WF-\eta_{1}|\right|}_{F}^{2}, \end{array}$$

where $I_{+}(X)=\{\begin{array}{cc} 0 & X\geq 0\\ \infty & \text{otherwise} \end{array},$ *η*_1_ is the Lagrange multiplier, and *μ*_1_ is fixed positive parameter. The iterative scheme became


(9) $$\begin{array}{c} F^{\left(k+1\right)}=\arg\min_{F}L(F,X^{\left(k\right)},U^{\left(k\right)},\eta_{1}^{\left(k\right)})\\ U^{\left(k+1\right)}=\arg\min_{U}L(F^{\left(k+1\right)},X^{\left(k\right)},U,\eta_{1}^{\left(k\right)})\\ X^{\left(k+1\right)}=\arg\min_{X}L(F^{\left(k+1\right)},X,U^{\left(k+1\right)},\eta_{1}^{\left(k\right)})\\ \eta_{1}^{\left(k+1\right)}=\eta_{1}^{\left(k\right)}-(U^{\left(k+1\right)}-WF^{\left(k+1\right)}), \end{array}$$

where *k* indexes the iteration steps.

The objective function of the *F*-subproblem was in a form of a summation of three least squares terms. Hence, it had a closed-form solution


(10) $$F^{\left(k+1\right)}={\left(P^{T}P+\beta I+\mu_{1}W^{T}W\right)}^{-1}(P^{T}B+\beta X^{\left(k\right)}(\Lambda K+K_{E}^{C})+W^{T}(U^{\left(k\right)}-\eta_{1}^{\left(k\right)})).$$

In practice, we solved this using conjugate gradient algorithm instead of directly computing the matrix inverse, which was computationally challenging.

The *U*-subproblem was a soft shrinkage problem and can be solved for each pixel independently. The closed form solution is


(11) $$u_{i}^{\left(k+1\right)}=\max\left\{Wf_{i}^{\left(k\right)}+{\left(\eta_{1}^{\left(k\right)}\right)}_{i}-\frac{\alpha_{1}}{\mu_{1}},0\right\}\text{for }i=1,\ldots ,M.$$

As for the subproblem of *x*, the objective function is a least square term with the *l_1_* regularization and nonnegative constraint. There is no closed-form solution, and we have to solve it in an inner iteration scheme. Similar to Eq. (6), we used ADMM to solve this *X*-subproblem by introducing another auxiliary variable *Y*. The corresponding augmented Lagrangian function now becames


(12) $$L_{inner}(X,Y,\eta_{2})=\frac{\beta }{2}{\left||F-X(\Lambda K+K_{E}^{C})|\right|}_{F}^{2}+\alpha_{2}\sum_{j=1}^{M}{\left||y_{i}|\right|}_{1}+I_{+}(Y)+\frac{\mu_{2}}{2}{\left||Y-X-\eta_{2}|\right|}_{F}^{2},$$

where *η*_2_ is the Lagrange multiplier and *μ*_2_ is fixed positive parameter. Here, the iteration scheme was to alternatively update three variables:


(13) $$\begin{array}{c} X^{\left(p+1\right)}=\arg\min_{X}L_{inner}(X,Y^{\left(p\right)},\eta_{2}^{\left(p\right)})\\ Y^{\left(p+1\right)}=\arg\min_{Y}L_{inner}(X^{\left(p+1\right)},blackY,\eta_{2}^{\left(p\right)})\\ \eta_{2}^{\left(p+1\right)}=\eta_{2}^{\left(p\right)}-(Y^{\left(p+1\right)}-X^{\left(p+1\right)}), \end{array}$$

where the superscript *p* indexes the inner loop. Denote , the closed-form solution of *X* and *Y* were


(14) $$\begin{array}{c} X^{\left(p+1\right)}={\left(AA^{T}+\mu_{2}I\right)}^{-1}(AF^{\left(k+1\right)}+\mu_{2}(Y^{\left(p\right)}-\eta_{2}^{\left(p\right)}))\\ Y^{\left(p+1\right)}=\max\left\{X^{\left(p+1\right)}+\eta_{2}^{\left(p\right)}-\frac{\alpha_{2}}{\mu_{2}},0\right\}. \end{array}$$

The convergence of the proposed algorithm is guaranteed (19). The iterative process of the algorithm is summarized in **Algorithm 1**.

**Algorithm 1** ADMM algorithm Eq. (6)

**Input**: projection data *B*, dictionary Λ, *K*, and $K_{M}^{C}$

**Parameters**: *μ*_1_, *μ*_2_, *α*_1_, *α*_2_, *β*, tolerance ∈, kMax, and pMax

**Initialize**: *F*, *X*, *U*, *Y*, and *k*, *p* = 0

**while** *k* < kMax or ||*F* ^(^ *^k^* ^)^ – *F* ^(^ *^k–1^* ^)^||_2_/||*F* ^(^ *^k^* ^)^||_2_ >∈ **do**


$$\begin{array}{c} F^{\left(k+1\right)}={\left(P^{T}\;P+\beta I+\mu_{1}W^{T}W\right)}^{-1}(P^{T}B+\beta X^{\left(k\right)}(\Lambda K+K_{E}^{C})\\ +W^{T}(U^{\left(k\right)}-\eta_{1}^{\left(k\right)}))\\ u_{i}^{\left(k+1\right)}=\max\left\{Wf_{i}^{\left(k\right)}+{\left(\eta_{1}^{\left(k\right)}\right)}_{i}-\frac{\alpha_{1}}{\mu_{1}},0\right\}\text{ for }i=1,\ldots ,M \end{array}$$

**while** *p* < pMax or ||*X* ^(^ *^k^* ^)^ – *X* ^(^ *^k–1^* ^)^||_2_/||*X* ^(^ *^k^* ^)^||_2_ >∈ **do**


$$\begin{array}{c} X^{\left(p+1\right)}={\left(AA^{T}+\mu_{2}I\right)}^{-1}(AF^{\left(k+1\right)}+\mu_{2}(Y^{\left(p\right)}-\eta_{2}^{\left(p\right)}))\\ Y^{\left(p+1\right)}=\max\left\{X^{\left(p+1\right)}+\eta_{2}^{\left(p\right)}-\frac{\alpha_{2}}{\mu_{2}},0\right\}\\ \eta_{2}^{\left(p+1\right)}=\eta_{2}^{\left(p\right)}-(Y^{\left(p+1\right)}-X^{\left(p+1\right)})\\ p=p+1 \end{array}$$

**end while**

**return** *X* ^(^ *^k^* ^+ 1)^ = *X* ^(^ *^p^* ^)^


$$\begin{array}{c} \eta_{1}^{\left(k+1\right)}=\eta_{1}^{\left(k\right)}-(U^{\left(k+1\right)}-WF^{\left(k+1\right)})\\ k=k+1\;\;\text{and }p=\;0 \end{array}$$

**end while**

**return** *F* ^*^ = *F* ^(^ *^k^* ^)^ and *X* ^*^ = *X* ^(^ *^k^* ^)^

### 2.3 Dictionary

In our previous study (14), the dictionary was constructed with ECs of 71 human tissues of a reference human listed in a previous publication (20). In this study, to handle the image reconstruction problem in the presence of iodine element, we expanded the dictionary by adding one more tissue with 100% iodine element. Note that using a linear combination of this iodine tissue and other tissues in the dictionary, we can express materials with any iodine concentrations. Additionally, we removed Ca component from the dictionary. In clinical practice, iodine contrast agent only exists in soft tissue material, where the amount of Ca component is expected to be negligibly small.

### 2.4 Evaluations

**Figure 1:**
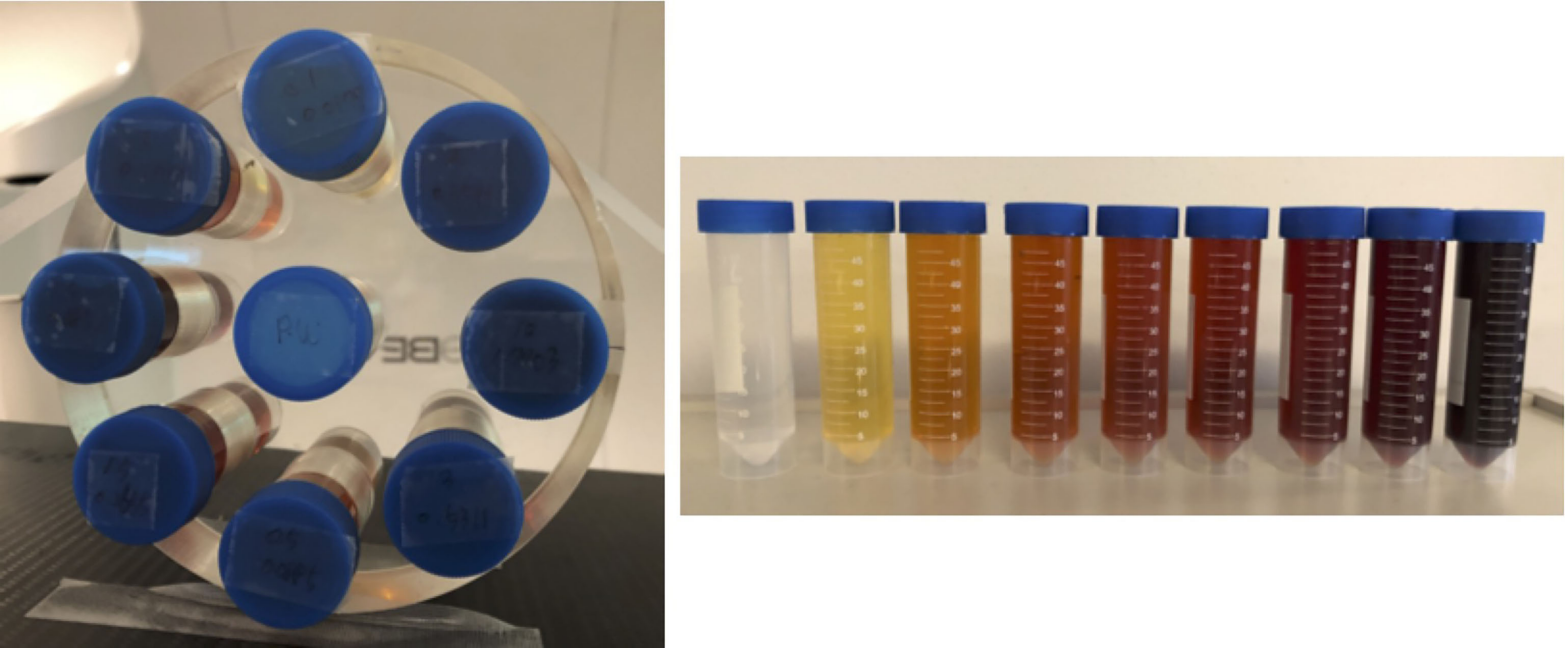
Phantom used in this study with inserts of different concentrations of iodine.

We performed both simulation studies and experimental validations using a phantom with inserts containing water and iodine solution (175 mgI/ml) of different concentrations, see **Figure 1**. In the experimental studies, the physical phantom was made of acrylic. Its diameter was 20 cm. Nine inserts were placed inside the phantom with one at the phantom center and eight evenly placed on the periphery with their centers on a circle of a radius of 7.5 cm. Each insert was a 50-ml plastic lab tube with a diameter of 3 cm. The solution for the insert at the phantom center was pure water. The other eight inserts contained iodine solution with concentrations of 0.1%, 0.5%, 1.0%, 1.5%, 2.0%, 3.0%, 5.0%, and 10% in weight (%*w*/*w*).

The hardware platform used in this study was the on-board imaging system of a Varian TrueBeam LINAC (Varian Medical System, Palo Alto, CA, USA). The source-to-isocenter distance was 100 cm, and the source-to-detector distance was 150 cm. We programmed the CBCT system to implement the kVp switching scanning protocol using its developer mode controlled by a customized “xml” file. We scanned the phantom to acquire x-ray projections. In a CBCT scan with 600 projections evenly distributed in a full gantry rotation, the energy levels were set to sequentially cycle through 80, 100, and 120 kVp. To ensure future clinical translation of the develop method, we employed x-ray beams in the 80–120-kVp range that are commonly available in clinical CT scanners. We also attempted to spread the kVp values to allow the largest possible spectrum separation, which is beneficial for material decomposition. These considerations led to the selected 80-, 100-, and 120-kVp beams. The tube currents for these three kVp levels were 1.4, 0.8, and 0.5 mAs, respectively. These mAs levels were empirically chosen. A relatively lower mAs was used for the channel with a higher kVp level to make the noise level approximately similar among different energy channels.

In the simulation studies, we constructed a digital phantom with the same dimension as the physical phantom. However, we replaced the background acrylic material with pure water for simplicity. The digital phantom was then voxelized with a voxel size of 1 mm^3^. We defined the material composition and density of all voxels based on known phantom information. x-ray projections of the digital phantom was then calculated following the same kVp switching scheme as in the actual experiment using our in-house developed Monte Carlo simulation tool (21). All other settings in the simulation matched those of the experiment.

In both simulation and experimental studies, we first reconstructed the MEER-CBCT images using Algorithm 1. To benchmark our method, we compared the reconstruction results against those of the clinical standard Filtered Back Projection (FBP) reconstruction method and those of iterative reconstruction method using single energy data. For FBP reconstruction, projections at 80, 100, and 120 kVps were simulated/acquired individually with 600 projections covering a whole gantry rotation to allow sufficient number of projections to reduce streak artifacts. This means that the comparison actually favored the FBP reconstruction case, as in the MEER-CBCT case, each energy only had one-third number of x-ray projections. For iterative reconstruction with single energy, the setup was the same as MEER-CBCT. The algorithm solved the problem in Eq. (1) with *β* = 0 to determine images *F*. This setting essentially treated the three energy channels independently and ignored the interchannel relationship expressed in Eq. (4).

To quantitatively evaluate the results, we considered two metrics. The first one was contrast enhancement defined as ${\bar{x}}_{\text{roi}}-{\bar{x}}_{\text{bg}},$ where ${\bar{x}}_{\text{roi}}$ is the mean value of the image intensity of a region of interest (ROI) and ${\bar{x}}_{\text{bg}}$ is the mean value of the image intensity of the background. The ROIs were selected as square regions within the eight circular inserts at the periphery region, and the background was the square region in the insert located at the center of the phantom. In the experimental study, the ROI selection was carefully performed to avoid bubbles in each insert. We used this metric to measure contrast enhancement caused by the iodine contrast agent. The second metric was contrast-to-noise ratio (CNR), a key quantity characterizing the detectability of an object (22). CNR was defined as


(15) $$\text{CNR}=\frac{2{\bar{x}}_{roi}-{\bar{x}}_{bg}}{\sqrt{\sigma_{roi}^{2}+\sigma_{bg}^{2}}},$$

where σ_roi_ and σ_bg_ are the standard deviations of image intensity at the ROI and the background, respectively. Additionally, since we know the ground truth iodine concentrations, we also evaluated errors of iodine concentrations derived by MEER-CBCT.

All the numerical computations analyzing results were conducted on a desktop with CPU (Intel i7-6700, 3.4 GHz) and MATLAB 9.2 (R2017a).

## 3 Results

### 3.1 Convergence and Parameter Sensitivity Analysis

**Figure 2:**
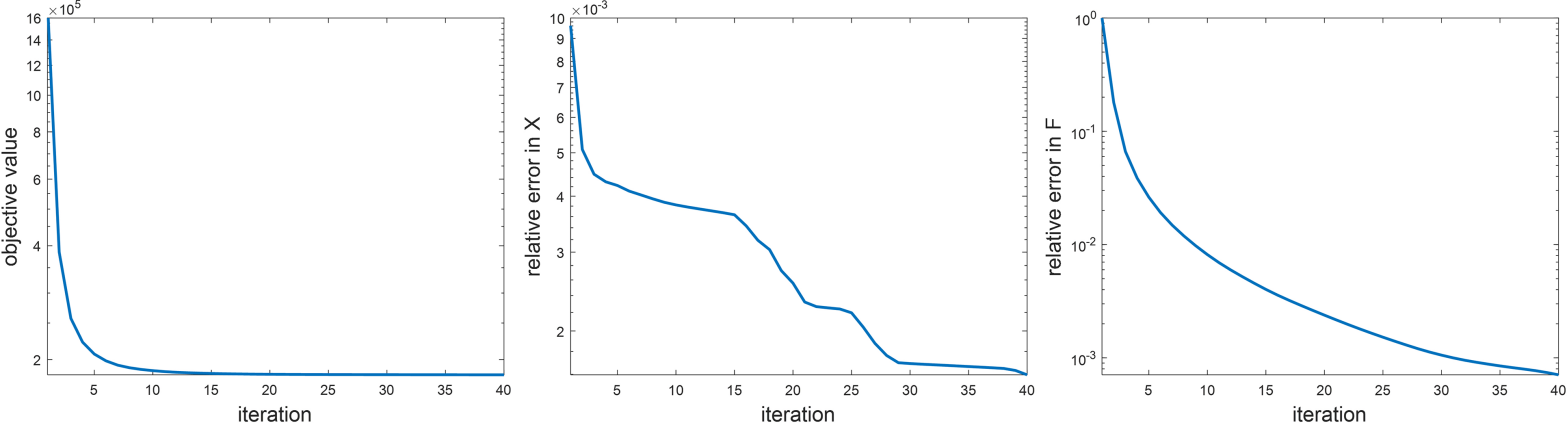
Empirical analysis on algorithm convergence. Left: objective value, middle: ||*X* ^(^ *^k^* ^)^ – *X* ^(^ *^k^* ^– 1)^||_2_/||*X* ^(^ *^k^* ^)^||_2_, right: *F* ^(^ *^k^* ^)^ – *F* ^(^ *^k^* ^–1)^||_2_/*F* ^(^ *^k^* ^)^||_2_.

Before presenting the image reconstruction results, we first show the convergence property of the numerical algorithm and results on sensitivity analysis of parameters in the algorithm. We empirically demonstrated the convergence of the proposed ADMM algorithm in **Figure 2**. Specifically, we examined the objective value in Eq. (6). We also studied the relative change in the restored images *F* and the variable *x* that was introduced to convexify the optimization problem between two successive iteration steps during the iteration process. Here, we only considered the simulation study. All the three quantities showed monotonically decaying trends. The objective function saturated at the end, indicating that the iteration reached convergence. We stopped the iteration at the step number 40, where the objective function value did not decrease significantly any further, and the relative changes of *x* and *F* were less than 10^-2^ and 10^-3^, respectively.

**Figure 3:**
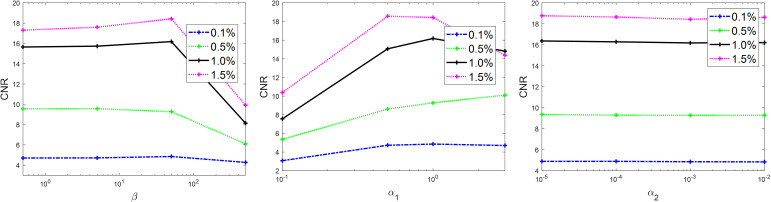
Sensitivity analysis on *β* (left), α_1_ (middle), and α_2_ (right).

There are multiple parameters in **Algorithm 1**, whose values are expected to affect the final results. Hence, it is important to study the sensitivity of the results to these parameters and select their optimal values. There are five parameters in **Algorithm 1**, *β*, *α*_1_, *α*_2_, *μ*_1_, and *μ*_2_. The two parameters *μ*_1_ and *μ*_2_ are expected to only affect algorithm convergence rate, but not the results. Therefore, we studied the sensitivity of parameters *β*, *α*_1_, and *α*_2_. It would be computationally challenging to scan the entire range of these parameters. Hence, we first sampled a few combinations of these parameters in the possible value range and gradually adjusted the parameters in a trial-and-error way to determine the optimal parameter set that yielded the highest CNR of different inserts in the reconstructed image with 80 kVp. After that, we fixed two parameters at their optimal values and studied the dependence of CNR as a function of the third parameter. Similar to the convergence study, the simulation case was used here. The results are shown in **Figure 3**. Based on this study, the optimal parameter values were *β* = 50 and *α*_1_ = 1.0. The CNR was found to be not sensitive to *α*_2_, and we set it to *α*_2_ = 0.001 in this study. These parameter values were used in subsequent image reconstruction studies.

### 3.2 Reconstruction Results

**Figure 4:**
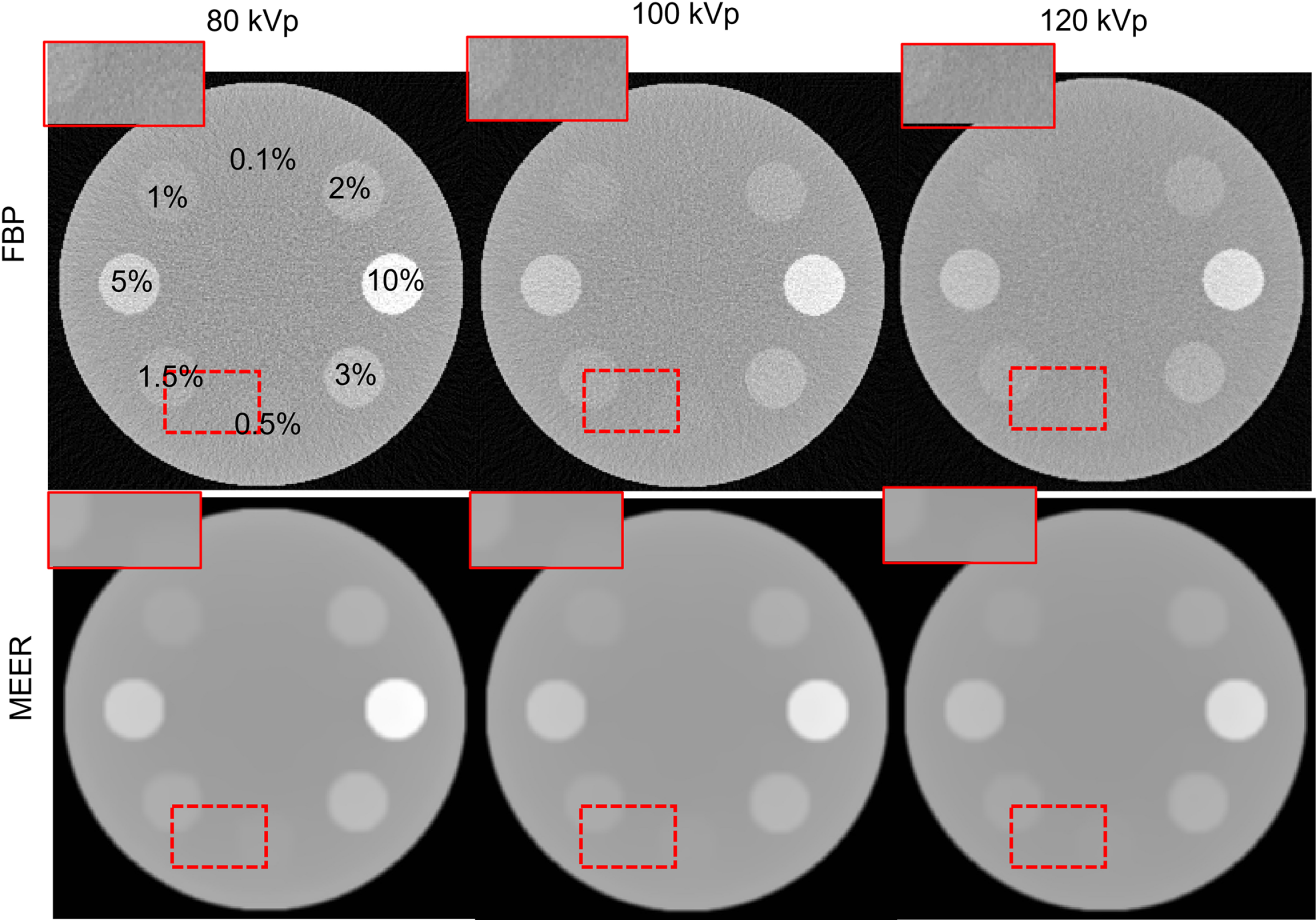
Reconstructed CT images in the simulation study. Dashed rectangles indicate zoomed in view regions. The CT images are displayed at a window of [-500, 500 HU].

**Figure 5:**
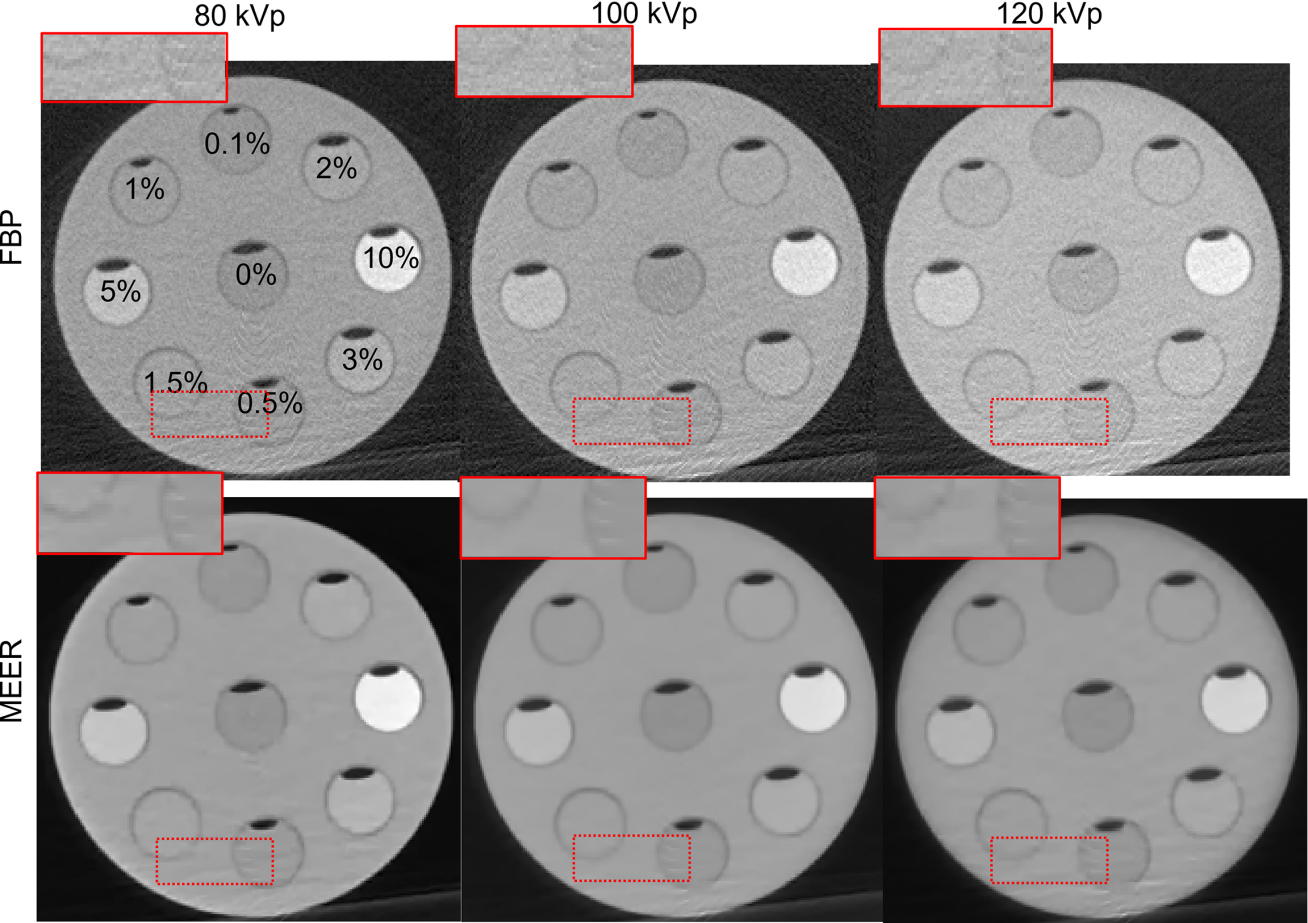
Reconstructed CT images in experimental study. Dashed rectangles indicate zoomed in view regions. The CT images are displayed at a window of [-500, 500] HU.

**Figures 4** and **5** present the reconstructed MEER-CBCT images in the simulation and the experimental studies, respectively. The first and the second rows in each figure are images of the phantom images reconstructed by the FBP algorithm and the MEER-CBCT algorithm, and columns are for different kVps. Comparing the results using the FBP reconstruction method, the images produced by the MEER-CBCT method achieved improved image quality, as indicated by visually reduced noise level, which can be ascribed to the inclusion of regularization terms in spatial and energy dimension in the reconstruction process.

**Figure 6:**
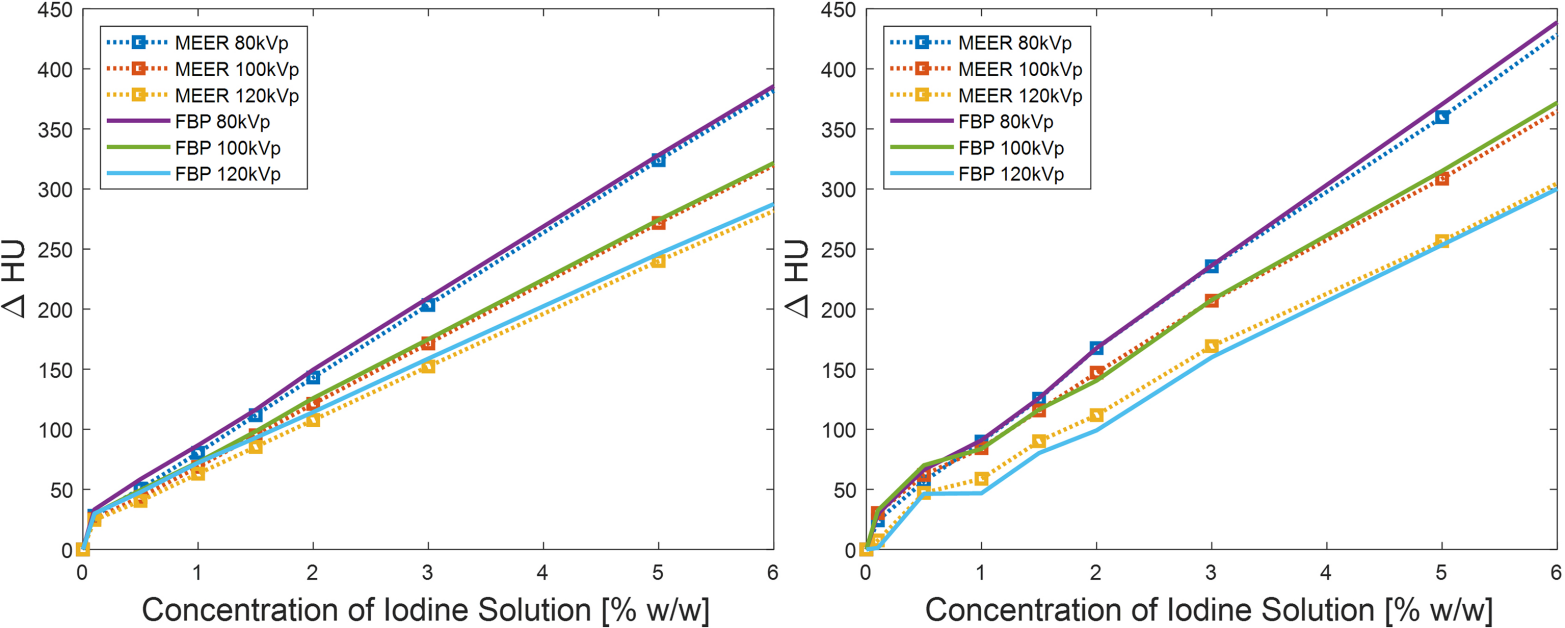
Comparison of contrast enhancements between our method and FBP reconstruction method in simulation study (left) and experimental study (right). Result of 10% iodine concentration not displayed to focus on relevant data range.

For quantitative comparison, we first present the results of contrast enhancement in **Figure 6**. As expected, the contrast enhancement increased approximately linearly with respect to the iodine agent concentration. The contrast enhancement became higher for lower kVp levels due to increased photoelectric interactions at low-energy range. The enhancement levels in the images reconstructed by the FBP algorithm and by the MEER-CBCT algorithm generally agreed with each other, indicating that the use of regularization terms in our method did not suppress image contrast.

**Figure 7:**
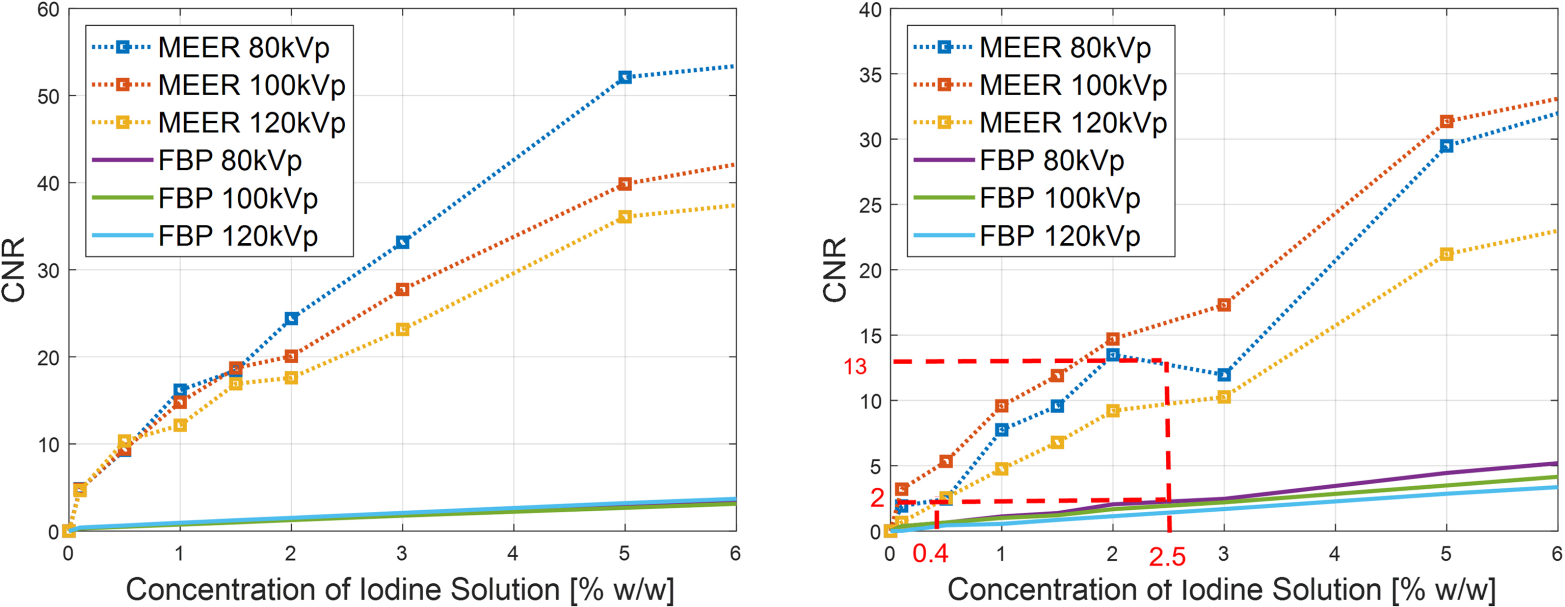
Comparison of CNRs between our method and FBP reconstruction method in simulation study (left) and experimental study (right). Result of 10% iodine concentration not displayed to focus on relevant data range.

**Figure 7** presents the results of CNRs. As expected, CNRs increased with iodine agent concentration and reduced kVp levels. In both simulation and experimental studies, for a given concentration of the iodine solution, the corresponding CNR obtained by our method was significantly higher than that of the FBP reconstruction method. For instance, the CNR of MEER-CBCT was approximately 6.5 times of that of the FBP method at 2.5% iodine contrast concentration in the experimental study (13.0 and 2.0, respectively). This can be ascribed to the use of image domain regularization, as well as the correlation among images at different kVps that was made possible by Eq. (4).

We considered CNR = 2.0 as the threshold of tumor detectability. Based on the experimental study [**Figure 7** (right)], the case with 2.5% of iodine contrast solution concentration yielded this level of CNR (averaged over all energy channels) for the conventional FBP reconstruction method. At this concentration level, **Figure 6** indicates that the averaged contrast enhancement over all energy channels was about ~170 HU. In clinical practice, the reported contrast between liver tumor and normal liver varies in the range of ~50 HU to ~300 HU depending on specific protocols of CT scan and contrast injection (23–25). The enhancement at this 2.5% iodine contrast case fell in this range. Compared with the FBP reconstruction method, MEER-CBCT achieved the same CNR level of 2.0 at ~0.4% of iodine solution concentration, about 6.3 times reduction of concentration.

**Figure 8:**
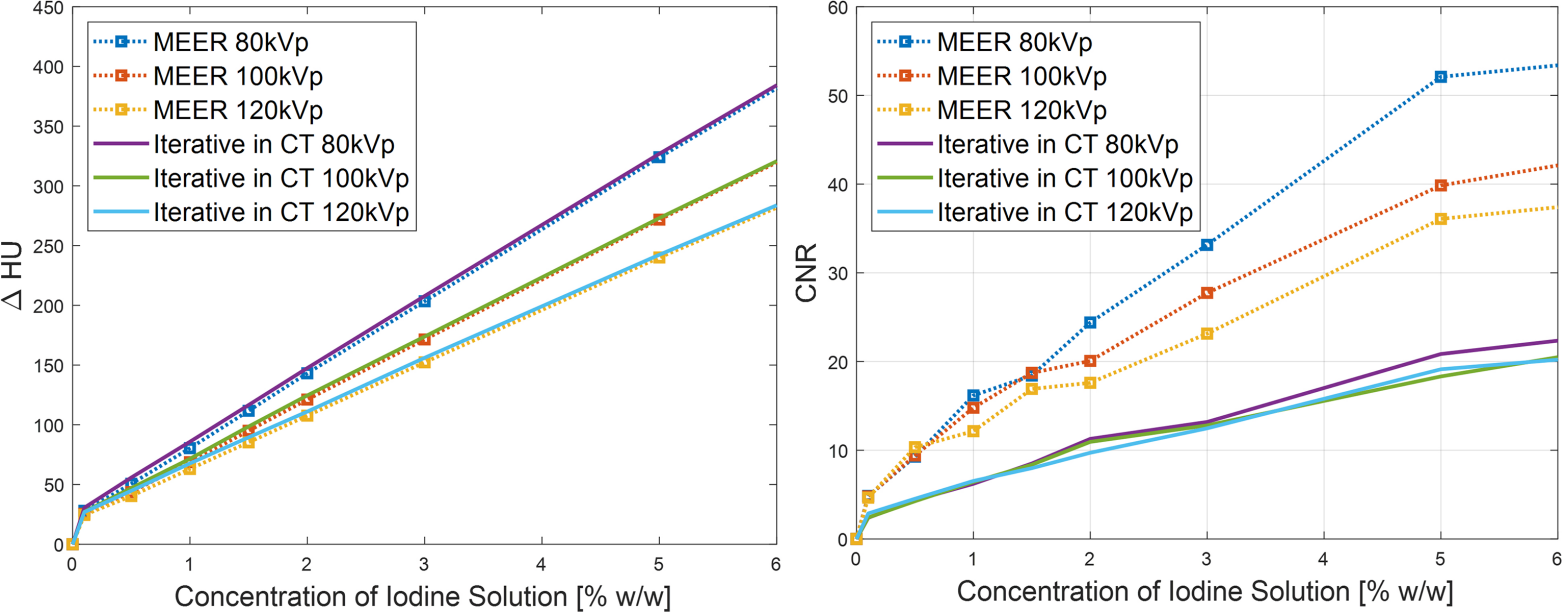
Comparison of CNR (left) and contrast enhancement (right) between MEER-CBCT and images reconstructed by iterative algorithm with each energy channel independently in simulation study.

**Figure 8** presents comparisons of contrast enhancement results and CNRs between MEER-CBCT images and those reconstructed by iterative reconstruction algorithm for each energy channel independently in the simulation case. It was observed that the two methods maintained the same level of contrast enhancement. MEER-CBCT was able to improve CNR by approximately a factor of 2 because of the incorporation of interenergy relationship.

**Figure 9:**
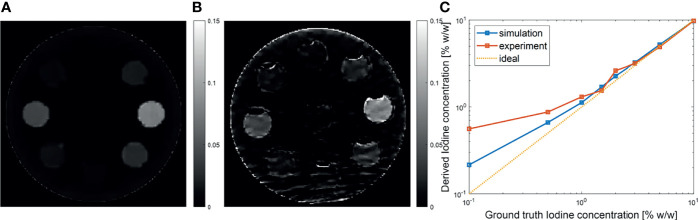
**(A, B)** Iodine concentration images in simulation and experimental studies. **(C)** Derived iodine concentration as a function of ground truth value. Dashed diagonal line indicates the ideal situation.

One advantage of MEER-CBCT algorithm is the capability of resolving iodine contrast distribution. **Figure 9A, B** presents the computed iodine concentration image in the simulation and the experimental studies, respectively. We plotted the iodine concentration in each insert over the known ground truth value in **Figure 9C**. The results derived by MEER-CBCT agreed well with ground truth values in general. The deviation from ground truth started to appear at low iodine concentration cases. Note the plot is on a log-log scale. The mean relative errors for the simulation and the experimental studies were 15% and 20%.

## 4 Discussion

One important aspect for clinical application of CBCT for radiotherapy image guidance is x-ray radiation dose, as excessive radiation dose can increase risk of secondary cancer. In the MEER-CBCT approach, we acquired x-ray projection data in a single rotation *via* the kVp switching approach. The radiation dose is hence expected to be approximately the average of the radiation dose of CBCT scans of each individual kVp scan, which is considered acceptable.

One contribution of this study is convexifying the reconstruction model presented in our previous work (14). The advantages of this approach included a unique solution to the optimization problem, and more importantly, a numerical algorithm that can solve the problem more efficiently. In the simulation study, our algorithm was 3.5 times faster than the one used to solve the nonconvex model in (14) with the same performance. Meanwhile, because of the convex nature, our model does not rely on the choice of initial guess, which is a favorable feature for practical applications.

Despite the exciting results in terms of achieving the same level of CNR as the FBP algorithm with substantially reduced iodine dose, the current study has a few limitations. First, the study was performed using a relatively small size phantom, where the impact of x-ray scatter was not significant. It is well known that x-ray scatter is a major concern affecting quantitative accuracy of CBCT imaging due to the large x-ray illumination field and detector size (26). In the liver site, the large body size would increase scatter component and reduce primary component of the x-ray beam at the detector, making the impact of scatter more profound than that of the phantom case. Hence, future challenges include proper removal of scatter before CBCT reconstruction. Over the years, advanced hardware-based or computation-based scatter correction methods have been successfully developed (26–28). These methods could be used to preprocess the x-ray projection data before using our algorithm for MEER-CBCT reconstruction.

Second, many factors affecting contrast agent visualization in CBCT images have not been considered in this initial study, which hence pose challenges to translate our method to clinical practice. The first factor is kinetic behavior of contrast enhancement of liver tumor. In fact, the liver tumor contrast enhancement shows a complex kinetic behavior occurring during a time interval with a length comparable with the CBCT data acquisition time (23). The CBCT acquisition has to be performed at a proper time after contrast injection, and the data acquisition time would average the contrast enhancement level. The second factor is organ motion. Liver is subject to respiratory motion. This motion during the CBCT scan, if unaddressed, would further blur the resulting images and diminish the contrast enhancement in the reconstructed images. We are in the process of developing a liver phantom with a realistic contrast enhancement mechanism and motion to study the impact of these factors. Novel image reconstruction techniques, such as reconstruction algorithms with temporal dimension included (29) or using deep learning (30), may be potentially employed to overcome these challenges.

## 5 Conclusion

To improve the detectability of iodine contrast agent in CBCT for image guidance of liver cancer radiotherapy, we developed a MEER-CBCT framework that acquired x-ray projections in a kVp switching scan on a conventional CBCT platform of a LINAC. MEER-CBCT image reconstruction method simultaneously reconstructed x-ray attenuation images at all kVp levels, the image of rED and images of EC. The composition of each voxel was subject to a constraint of a sparse representation of materials in a dictionary containing typical human tissues and iodine. We converted the nonlinear formalism of MEER-CBCT reconstruction problem to a linear form to ease the burden solving this problem. In both simulation and experimental studies, MEER-CBCT achieved similar contrast enhancement as the clinical standard FBP reconstruction method but significantly higher CNR. At 2.5% iodine solution concentration, FBP method achieved ~170 HU enhancement and CNR of ~2, considered the acceptable CNR for successful liver tumor visualization. MEER-CBCT yielded the same CNR but at ~6.3 times lower iodine concentration of 0.4%.

## Data Availability Statement

The raw data supporting the conclusions of this article will be made available by the authors upon request.

## Author Contributions

XJ, CS, and MY contributed to the study concepts and study design. CW and HJ contributed to data acquisition and image reconstructions. CW, HJ, and CS contributed to the data analyses and interpretation. All authors contributed to the manuscript preparation and editing. All authors contributed to the article and approved the submitted version.

## Funding

This work was supported in part by the National Institutes of Health (Grant Numbers R37CA214639 and R01CA227289).

## Conflict of Interest

The authors declare that the research was conducted in the absence of any commercial or financial relationships that could be construed as a potential conflict of interest.

## Publisher’s Note

All claims expressed in this article are solely those of the authors and do not necessarily represent those of their affiliated organizations, or those of the publisher, the editors and the reviewers. Any product that may be evaluated in this article, or claim that may be made by its manufacturer, is not guaranteed or endorsed by the publisher.

## References

[1] Bujold A Craig T Jaffray D Dawson LA. Image-Guided Radiotherapy: Has it Influenced Patient Outcomes? Semin Radiat Oncol (Elsevier) (2012) 22:50–61. doi: 10.1016/j.semradonc.2011.09.001 22177878

[2] Jaffray DA Siewerdsen JH Wong JW Martinez AA. Flat-Panel Cone-Beam Computed Tomography for Image-Guided Radiation Therapy. Int J Radiat Oncol Biol Phys (2002) 53:1337–49. doi: 10.1016/S0360-3016(02)02884-5 12128137

[3] Eccles CL Tse RV Hawkins MA Lee MT Moseley DJ Dawson LA. Intravenous Contrast-Enhanced Cone Beam Computed Tomography (Ivcbct) of Intrahepatic Tumors and Vessels. Adv Radiat Oncol (2016) 1:43–50. doi: 10.1016/j.adro.2016.01.001 28740872 PMC5506729

[4] Li N Zarepisheh M Uribe-Sanchez A Moore K Tian Z Zhen X. Automatic Treatment Plan Re-Optimization for Adaptive Radiotherapy Guided With the Initial Plan Dvhs. Phys Med Biol (2013) 58:8725. doi: 10.1088/0031-9155/58/24/8725 24301071

[5] Yan D Vicini F Wong J Martinez A. Adaptive Radiation Therapy. Phys Med Biol (1997) 42:123. doi: 10.1088/0031-9155/42/1/008 9015813

[6] McCollough CH Leng S Yu L Fletcher JG. Dual-And Multi-Energy Ct: Principles, Technical Approaches, and Clinical Applications. Radiology (2015) 276:637–53. doi: 10.1148/radiol.2015142631 PMC4557396 26302388

[7] McCollough CH Boedeker K Cody D Duan X Flohr T Halliburton SS. Principles and Applications of Multienergy Ct: Report of Aapm Task Group 291. Med Phys (2020) 47:e881–912. doi: 10.1002/mp.14157 32215937

[8] Niu T Dong X Petrongolo M Zhu L. Iterative Image-Domain Decomposition for Dual-Energy Ct. Med Phys (2014) 41:041901. doi: 10.1118/1.4866386 24694132

[9] Wang T Zhu L. Dual Energy Ct With One Full Scan and a Second Sparse-View Scan Using Structure Preserving Iterative Reconstruction (Spir). Phys Med Biol (2016) 61:6684. doi: 10.1088/0031-9155/61/18/6684 27552793 PMC6200581

[10] Zbijewski W Gang G Xu J Wang A Stayman JW Taguchi K. Dual-Energy Cone-Beam Ct With a Flat-Panel Detector: Effect of Reconstruction Algorithm on Material Classification. Med Phys (2014) 41:021908. doi: 10.1118/1.4863598 24506629 PMC3977791

[11] Lee D Lee J Kim H Lee T Soh J Park M. A Feasibility Study of Low-Dose Single-Scan Dual-Energy Cone-Beam Ct in Many-View Under-Sampling Framework. IEEE Trans Med Imaging (2017) 36:2578–87. doi: 10.1109/TMI.2017.2765760 29192887

[12] Li B Shen C Chi Y Yang M Lou Y Zhou L. Multienergy Cone-Beam Computed Tomography Reconstruction With a Spatial Spectral Nonlocal Means Algorithm. SIAM J Imaging Sci (2018) 11:1205–29. doi: 10.1137/17M1123237 PMC6173488 30298098

[13] Cassetta R Lehmann M Haytmyradov M Patel R Wang A Cortesi L. Fast-Switching Dual Energy Cone Beam Computed Tomography Using the on-Board Imager of a Commercial Linear Accelerator. Phys Med Biol (2020) 65:015013. doi: 10.1088/1361-6560/ab5c35 31775131 PMC7043019

[14] Shen C Li B Lou Y Yang M Zhou L Jia X. Multienergy Element-Resolved Cone Beam Ct (Meer-Cbct) Realized on a Conventional Cbct Platform. Med Phys (2018) 45:4461–70. doi: 10.1002/mp.13169 PMC6553481 30179261

[15] Jia X Dong B Lou Y Jiang SB. Gpu-Based Iterative Cone-Beam Ct Reconstruction Using Tight Frame Regularization. Phys Med Biol (2011) 56:3787. doi: 10.1088/0031-9155/56/13/004 21628778

[16] Cai JF Osher S Shen Z. Split Bregman Methods and Frame Based Image Restoration. Multiscale Model Simul (2009) 8:337–69. doi: 10.1137/090753504

[17] Rutherford R Pullan B Isherwood I. Measurement of Effective Atomic Number and Electron Density Using an Emi Scanner. Neuroradiology (1976) 11:15–21. doi: 10.1007/BF00327253 934468

[18] Shen C Li B Chen L Yang M Lou Y Jia X. Material Elemental Decomposition in Dual and Multi-Energy Ct *via* a Sparsity-Dictionary Approach for Proton Stopping Power Ratio Calculation. Med Phys (2018) 45:1491–503. doi: 10.1002/mp.12796 PMC5904041 29405340

[19] Boyd S Parikh N Chu E Peleato B Eckstein J. Distributed Optimization and Statistical Learning *via* the Alternating Direction Method of Multipliers. Found Trends ® Mach Learn (2011) 3:1–122. doi: 10.1561/9781601984616

[20] Schneider W Bortfeld T Schlegel W. Correlation Between Ct Numbers and Tissue Parameters Needed for Monte Carlo Simulations of Clinical Dose Distributions. Phys Med Biol (2000) 45:459. doi: 10.1088/0031-9155/45/2/314 10701515

[21] Jia X Yan H Cerviño L Folkerts M Jiang SB. A Gpu Tool for Efficient, Accurate, and Realistic Simulation of Cone Beam Ct Projections. Med Phys (2012) 39:7368–78. doi: 10.1118/1.4766436 PMC3523889 23231286

[22] Goenka AH Herts BR Dong F Obuchowski NA Primak AN Karim W. Image Noise, Cnr, and Detectability of Low-Contrast, Low-Attenuation Liver Lesions in a Phantom: Effects of Radiation Exposure, Phantom Size, Integrated Circuit Detector, and Iterative Reconstruction. Radiology (2016) 280:475–82. doi: 10.1148/radiol.2016151621 26937709

[23] Baron RL. Understanding and Optimizing Use of Contrast Material for Ct of the Liver. AJR. Am J Roentgenology (1994) 163:323–31. doi: 10.2214/ajr.163.2.8037023 8037023

[24] Guerrisi A Marin D Nelson R De Filippis G Di Martino M Barnhart H. Effect of Varying Contrast Material Iodine Concentration and Injection Technique on the Conspicuity of Hepatocellular Carcinoma During 64-Section Mdct of Patients With Cirrhosis. Br J Radiol (2011) 84:698–708. doi: 10.1259/bjr/21539234 21750137 PMC3473424

[25] Yanaga Y Awai K Nakaura T Namimoto T Oda S Funama Y. Optimal Contrast Dose for Depiction of Hypervascular Hepatocellular Carcinoma at Dynamic Ct Using 64-Mdct. Am J Roentgenology (2008) 190:1003–9. doi: 10.2214/AJR.07.3129 18356448

[26] Niu T Zhu L. Scatter Correction for Full-Fan Volumetric Ct Using a Stationary Beam Blocker in a Single Full Scan. Med Phys (2011) 38:6027–38. doi: 10.1118/1.3651619 PMC3215690 22047367

[27] Xu Y Bai T Yan H Ouyang L Pompos A Wang J. A Practical Cone-Beam Ct Scatter Correction Method With Optimized Monte Carlo Simulations for Image-Guided Radiation Therapy. Phys Med Biol (2015) 60:3567. doi: 10.1088/0031-9155/60/9/3567 25860299 PMC4409575

[28] Wang T Lei Y Fu Y Wynne JF Curran WJ Liu T. A Review on Medical Imaging Synthesis Using Deep Learning and its Clinical Applications. J Appl Clin Med Phys (2021) 22:11–36. doi: 10.1002/acm2.13121 PMC7856512 33305538

[29] Wang J Gu X. Simultaneous Motion Estimation and Image Reconstruction (Smeir) for 4d Cone-Beam Ct. Med Phys (2013) 40:101912. doi: 10.1118/1.4821099 24089914

[30] Shen C Nguyen D Zhou Z Jiang SB Dong B Jia X. An Introduction to Deep Learning in Medical Physics: Advantages, Potential, and Challenges. Phys Med Biol (2020) 65:05TR01. doi: 10.1088/1361-6560/ab6f51 PMC7101509 31972556

